# Large Individual Differences in Functional Connectivity in the Context of Major Depression and Antidepressant Pharmacotherapy

**DOI:** 10.1523/ENEURO.0286-23.2024

**Published:** 2024-06-06

**Authors:** Gwen van der Wijk, Mojdeh Zamyadi, Signe Bray, Stefanie Hassel, Stephen R. Arnott, Benicio N. Frey, Sidney H. Kennedy, Andrew D. Davis, Geoffrey B. Hall, Raymond W. Lam, Roumen Milev, Daniel J. Müller, Sagar Parikh, Claudio Soares, Glenda M. Macqueen, Stephen C. Strother, Andrea B. Protzner

**Affiliations:** ^1^Department of Psychology, University of Calgary, Calgary, Alberta T2N 1N4, Canada; ^2^Baycrest Health Sciences, Rotman Research Institute, Toronto, Ontario M6A 2E1, Canada; ^3^Child and Adolescent Imaging Research Program, University of Calgary, Calgary, Alberta T2N 1N4, Canada; ^4^Alberta Children’s Hospital Research Institute, University of Calgary, Calgary, Alberta T2N 1N4, Canada; ^5^Hotchkiss Brain Institute, University of Calgary, Calgary, Alberta T2N 1N4, Canada; ^6^Department of Radiology, University of Calgary, Calgary, Alberta T2N 1N4, Canada; ^7^Department of Psychiatry, Cumming School of Medicine, University of Calgary, Calgary, Alberta T2N 1N4, Canada; ^8^Mathison Centre for Mental Health Research and Education, University of Calgary, Calgary, Alberta T2N 14, Canada; ^9^Department of Psychiatry and Behavioural Neurosciences, McMaster University, Hamilton, Ontario L8S 4L8, Canada; ^10^Mood Disorders Program and Women’s Health Concerns Clinic, St. Joseph’s Healthcare, Hamilton, Ontario L8N 4A6, Canada; ^11^Department of Psychiatry, University of Toronto, Toronto, Ontario M5S 1A1, Canada; ^12^Institute of Medical Sciences, University of Toronto, Toronto, Ontario M5S 1A1, Canada; ^13^Centre for Mental Health, University Health Network, Toronto, Ontario M5G 2C4, Canada; ^14^Centre for Depression and Suicide Studies, Unity Health Toronto, Toronto, Ontario M5B 1W8, Canada; ^15^Krembil Research Institute, Toronto Western Hospital, Toronto, Ontario M5T 2S8, Canada; ^16^Department of Psychology, Neuroscience and Behaviour, McMaster University, Hamilton, Ontario L8S 4L8, Canada; ^17^Imaging Research Centre, St. Joseph’s Healthcare Hamilton, Hamilton, Ontario L8N 4A6, Canada; ^18^Department of Psychiatry, University of British Columbia, Vancouver, British Columbia V6T 2A1, Canada; ^19^Department of Psychiatry and Psychology, and Providence Care Hospital, Queen’s University, Kingston, Ontario K7L 3N6, Canada; ^20^Campbell Family Mental Health Research Institute, Centre for Addiction and Mental Health, Toronto, Ontario M5T 1R8, Canada; ^21^Department of Pharmacology and Toxicology, University of Toronto, Toronto, Ontario M5S 1A1, Canada; ^22^ Department of Psychiatry, University of Michigan, Ann Arbor, Michigan 48109; ^23^Department of Psychiatry, Queen’s University, Providence Care, Kingston, Ontario K7L 3N6, Canada; ^24^Department of Medical Biophysics, University of Toronto, Toronto, Ontario M5S 1A1, Canada

**Keywords:** fMRI, functional connectivity, individual differences, major depression, pharmacotherapy

## Abstract

Clinical studies of major depression (MD) generally focus on group effects, yet interindividual differences in brain function are increasingly recognized as important and may even impact effect sizes related to group effects. Here, we examine the magnitude of individual differences in relation to group differences that are commonly investigated (e.g., related to MD diagnosis and treatment response). Functional MRI data from 107 participants (63 female, 44 male) were collected at baseline, 2, and 8 weeks during which patients received pharmacotherapy (escitalopram, *N* = 68) and controls (*N* = 39) received no intervention. The unique contributions of different sources of variation were examined by calculating how much variance in functional connectivity was shared across all participants and sessions, within/across groups (patients vs controls, responders vs nonresponders, female vs male participants), recording sessions, and individuals. Individual differences and common connectivity across groups, sessions, and participants contributed most to the explained variance (>95% across analyses). Group differences related to MD diagnosis, treatment response, and biological sex made significant but small contributions (0.3–1.2%). High individual variation was present in cognitive control and attention areas, while low individual variation characterized primary sensorimotor regions. Group differences were much smaller than individual differences in the context of MD and its treatment. These results could be linked to the variable findings and difficulty translating research on MD to clinical practice. Future research should examine brain features with low and high individual variation in relation to psychiatric symptoms and treatment trajectories to explore the clinical relevance of the individual differences identified here.

## Significance Statement

Studies on major depression (MD) often investigate differences in brain function between groups (e.g., those with/without a diagnosis) to better understand this prevalent condition. Our study examines such group differences in the context of similarities across and differences between individuals. We found strong common and individually unique features of brain network organization, relative to surprisingly subtle features of diagnosis, treatment success, and sex assigned at birth. From the overall explained variation in brain connectivity, ∼50% was shared across everyone, while another 45% was unique to individuals. Only ∼5% could be attributed to group differences. Our results suggest that examining individual differences, and their potential clinical relevance, alongside group differences may bring us closer to improving clinical outcomes for MD.

## Introduction

Neuroimaging studies examining major depression (MD) have identified group differences between patients and controls, for different treatment outcomes, and in relation to biological sex/gender (Ruigrok et al., 2014; Dichter et al., 2015; Gudayol-Ferré et al., 2015; Brakowski et al., 2017; Labaka et al., 2018; Wheelock et al., 2019; Bangasser and Cuarenta, 2021). Less attention has been given to individual differences and commonalities across groups. In neurotypical individuals, common and stable individual features were the largest contributors to the variance in fMRI functional connectivity (FC) across sessions and tasks (Gratton et al., 2018). In addition, such stable individual features may relate to cognition and clinical symptoms (Finn et al., 2015; Gordon et al., 2018; D. Wang et al., 2020; Lynch et al., 2023). These findings have led to calls for nuanced examinations of individual differences and commonalities in psychiatric populations like MD (Etkin, 2019; Gratton et al., 2020).

Many studies have investigated group differences in FC in the context of MD and antidepressant treatment (Dichter et al., 2015; Gudayol-Ferré et al., 2015; Mulders et al., 2015; Brakowski et al., 2017). Connectivity features related to MD are most consistently identified across three major resting state networks; the default mode network (DMN), salience network (SN), and cognitive control network (CCN; Mulders et al., 2015; Brakowski et al., 2017). Similarly, connectivity features have been linked to treatment success, most commonly within the (anterior) DMN and between frontal and limbic regions (Dichter et al., 2015; Gudayol-Ferré et al., 2015). Despite such consistencies, differences across studies were also observed. Next to variable methodologies, patient heterogeneity is often cited as a potential reason for inconsistencies (Brakowski et al., 2017; Müller et al., 2017).

Some key sources of heterogeneity include clinical symptoms (Fried and Nesse, 2015), response to treatment (Rush et al., 2006), and biological sex, gender identity, and gender expression in clinical as well as brain features (Whiteford et al., 2013; Altemus et al., 2014; Labaka et al., 2018; Bangasser and Cuarenta, 2021). Though more research is needed to understand the complex biological, social, cultural, and socioeconomic causes underlying these differences, higher rates of MD have been observed in women compared with those in men (Whiteford et al., 2013) and the expression of MD symptoms differs between women and men (Altemus et al., 2014). Women may also respond better to SSRIs than men (Khan et al., 2005). Less is known about how these factors relate to individual differences in brain function.

The only other deep phenotyping study examining MD suggests that individual differences in salience network topology can be linked to depressive symptomatology and may confer depression risk (Lynch et al., 2023, preprint). Deep phenotyping in neurotypical samples also highlights stable individual differences that are greater in association networks such as the salience network and less in primary sensorimotor networks (Mueller et al., 2013; Chen et al., 2015; Gratton et al., 2018; Kong et al., 2019; Seitzman et al., 2019). Importantly, this body of work indicates that more data than typically used in neuroimaging studies of MD (e.g., 25–200 min instead of 5–10 min) are needed for stable individual estimates of FC (Laumann et al., 2015; Gordon et al., 2017; Gratton et al., 2020). Using longer fMRI scans to explore individual alongside group differences in the brain is especially relevant for MD, considering the hopes that neuroimaging will eventually support clinical decision-making for individual patients (Fu and Costafreda, 2013; Fonseka et al., 2018).

Here, we examined the relative contribution of sources of variance in neuroimaging data in the context of MD and its treatment and in relation to biological sex. We used multisite fMRI data from the CAN-BIND initiative, including 68 patients and 39 controls (Lam et al., 2016; Kennedy et al., 2019). Participants were scanned three times (baseline, 2, and 8 weeks; ∼30 min of fMRI data per scan) with patients receiving escitalopram after their baseline scan. We calculated the cross-correlation between individual- and session-specific FC matrices to assess similarities and differences within and across groups (controls vs patients, treatment responders vs nonresponders, female vs male participants), sessions (baseline, 2, and 8 weeks), and individuals, as well as the MD × session and MD × sex interactions. We also assessed where in the brain these effects were most prominent. We hypothesized that considerable variance would be explained by individual differences and similarities across all participants and sessions (common effect; Carbonell et al., 2011), with smaller contribution from diagnosis, treatment success, and sex assigned at birth.

## Materials and Methods

The data analyzed in this study were collected as part of the Canadian Biomarker Integration Network for Depression (CAN-BIND) initiative. A detailed report of the protocol (Lam et al., 2016) and primary outcomes (Kennedy et al., 2019) have been published elsewhere. We preregistered our analyses and hypotheses on the Open Science Framework (OSF; https://osf.io/79gv8/?view_only=9e105962ce4c4ebf8cf35393471a7b69) and report the changes that we made postregistration (see Transparent_Changes_Document.docx).

### Participants

The sample included in these analyses consisted of 107 participants, including 68 patients with a primary diagnosis of MD and 39 controls. From the larger CAN-BIND-1 dataset, we selected participants who had complete and high-quality neuroimaging data for the resting state, affective go/no-go, and monetary incentive delay tasks at all three fMRI recording sessions, to get the longest total recording times under similar conditions per participant. Sex was measured as the self-reported sex of the individual (based on biological reproductive functions) and/or the clinician's assignment based on a physical examination and was coded as binary (female or male; see limitations section for discussion on this). Using this definition, among patients and controls, respectively, 42 and 21 participants were female, while 26 and 18 were male. Participants were recruited at six sites in Canada, were 18–60 years of age, and spoke sufficient English for the completion of this study. Demographic information is reported in Table 1. Ethics approval was granted by local ethics committees, and all participants gave written informed consent.

**Table 1.** Demographic characteristics of female and male patients with MD and controls (mean ± SEM), and their statistical differences

	Patients with MD (*N* = 68)		Controls (*N* = 39)		Statistical difference
	Female (*N* = 42)	Male (*N* = 26)	Female (*N* = 21)	Male (*N* = 18)	
Age (years)	34.36 ± 1.81 (18–59)	38.46 ± 2.46 (18–59)	33.00 ± 2.59 (18–60)	34.89 ± 3.02 (21–57)	MD: *F*_(1,103)_ = 1.00, *p *= 0.32; sex: *F*_(1,103)_ = 1.48, *p *= 0.23; interaction: *F*_(1,103)_ = 0.65, *p *= 0.65
Education (years)	17.00 ± 0.26 (14–20)	17.38 ± 0.38 (14–20)	18.71 ± 0.38 (14–22)	18.22 ± 0.53 (13–22)	MD: *F*_(1,103)_ = 11.34, *p *= 0.001; sex: *F*_(1,103)_ = 0.02, *p *= 0.89; interaction: *F*_(1,103)_ = 1.34, *p *= 0.25
Race/ethnicity^a^	Black (2); East Asian (4); Jewish (1); Latin American/Hispanic (2); South Asian (1); Southeast Asian (2); White (34); Other (2)	Latin American/Hispanic (1); South Asian (2); Southeast Asian (1); White (22); Other (1)	East Asian (5); South Asian (3); Southeast Asian (2); White (12)	Latin American/Hispanic (1); South Asian (3); White (15)	NA
Site	CAM (4); MCU (7); QNS (2); UBC (20); UCA (9)	MCU (6); QNS (5); TGH (1); UBC (7); UCA (7)	CAM (2); MCU (3); QNS (6); TGH (2); UBC (4); UCA (4)	CAM (3); MCU (3); QNS (1); TGH (1); UBC (2); UCA (8)	NA
Handedness	Left (2); right (38); ambidextrous (2)	Left (5); right (21)	Left (2); right (19)	Left (5); right (10); ambidextrous (3)	NA
Current marital status	Never married (22); separated (1); married (9); divorced (4); domestic partnership (5); widowed (1)	Never married (14); separated (2); married (7); divorced (2); domestic partnership (1)	Never married (12); married (5); domestic partnership (4)	Never married (11); married (5); divorced (1); domestic partnership (1)	NA

MD, major depressive disorder; CAM, Centre for Addiction and Mental Health; MCU, McMaster University; UBC, University of British Columbia; TGH, Toronto General Hospital; QNS, Queen's University; UCA, University of Calgary, NA, not applicable.

a Participants were asked which of the presented race/ethnicity categories they most closely identified with. Categories were based on Canadian census questionnaires (Statistics Canada, 2006).

Diagnostic assessment using the Mini International Neuropsychiatric Interview (MINI; Sheehan et al., 1998) was performed by a psychiatrist to ensure MD participants met criteria for a major depressive episode according to the DSM-IV-TR, and entry level severity was confirmed using the Montgomery–Åsberg Depression Rating Scale (MADRS; Montgomery and Åsberg, 1979). All had MADRS scores >24 at enrollment, with their current episode lasting at least 3 months. Exclusion criteria for patients included meeting the diagnostic criteria for another psychiatric condition (except for anxiety), having a diagnosis of bipolar depression, experiencing psychotic symptoms in the current depressive episode, being at high risk for suicide or hypomanic switch, experiencing substance dependence in the last 6 months, being pregnant or breastfeeding, showing no response to four previous adequate pharmacotherapy interventions, and having a previous unfavorable response to the medications used in the study. Patients on antidepressant medications prior to the study went through a wash-out period equivalent to five half-lives or more. Controls had no psychiatric diagnosis (as assessed with the MINI), and participants from both groups were excluded if they had a history of neurological conditions, head trauma or other unstable medical conditions, or any contraindications to MRI.

After the baseline visit was completed, patients received escitalopram at an initial dose of 10 mg/day, increasing to 20 mg/day after 2 or 4 weeks unless contraindicated by side effects. Every 2 weeks, patients’ symptom scores were assessed using the structured interview guide (SIGMA) for the MADRS (Williams and Kobak, 2008). Participants whose MADRS scores decreased >50% from baseline to Week 8 were considered responders (*N* = 33), while participants whose MADRS scores decreased <50% (or increased) were considered nonresponders (*N* = 35). Demographic information for both groups is presented in Table 2.

**Table 2.** Clinical and demographic characteristics of female and male responders and nonresponders to antidepressant pharmacotherapy (mean ± SEM), and their statistical differences

	Responders (*N* = 33)		Nonresponders (*N* = 35)		Statistical difference
	Female (*N* = 19)	Male (*N* = 14)	Female (*N* = 23)	Male (*N* = 12)	
MADRS at baseline	28.16 ± 1.00	28.93 ± 1.07	30.39 ± 1.32	30.00 ± 1.33	Response: *F*_(1,64)_ = 1.67, *p *= 0.20; sex: *F*_(1,64)_ = 0.02, *p *= 0.88; interaction: *F*_(1,64)_ = 0.21, *p *= 0.65
MADRS at Week 2	18.11 ± 1.73	18.57 ± 1.73	24.48 ± 1.27	23.50 ± 1.78	Response: *F*_(1,64)_ = 11.61, *p *= 0.001; sex: *F*_(1,64)_ = 0.02, *p *= 0.88; interaction: *F*_(1,64)_ = 0.19, *p *= 0.66
MADRS at Week 8	8.21 ± 1.15	7.07 ± 1.49	20.61 ± 1.09	22.92 ± 1.35	Response: *F*_(1,64)_ = 119.95, *p *< 0.001; sex: *F*_(1,64)_ = 0.21, *p *= 0.65; Interaction: *F*_(1,64)_ = 1.79, *p *= 0.19
Age (years)	34 ± 2.49 (19–55)	38.36 ± 3.44 (18–59)	34.65 ± 2.63 (18–59)	38.58 ± 3.68 (20–59)	Response: *F*_(1,64)_ = 0.02, *p *= 0.89; sex: *F*_(1,64)_ = 1.84, *p *= 0.18; interaction: *F*_(1,64)_ < 0.01, *p *= 0.94
Education (years)	17.21 ± 0.43 (14–20)	17.29 ± 0.61 (14–20)	16.83 ± 0.32 (14–19)	17.50 ± 0.45 (16–20)	Response: *F*_(1,64)_ = 0.04, *p *= 0.85; sex: *F*_(1,64)_ = 0.68, *p *= 0.41; interaction: *F*_(1,64)_ = 0.43, *p *= 0.51
Race/ethnicity^a^	Black (1); East Asian (3); South Asian (1); White (15); Other	Latin American/Hispanic (1); South Asian (1); White (12); Other (1)	Black (1); East Asian (1); Jewish (1); Latin American/Hispanic (2); Southeast Asian (2); White (19); Other (1)	South Asian (1); Southeast Asian (1); White (10)	NA
Site	CAM (2); MCU (2); UBC (9); UCA (6)	MCU (3); QNS (3); TGH (1); UBC (4); UCA (3)	CAM (2); MCU (5); QNS (2); UBC (11); UCA (3)	MCU (3); QNS (2); UBC (3); UCA (4)	NA
Handedness	Left (2); right (16); ambidextrous (1)	Left (3); right (11)	Right (22); ambidextrous (1)	Left (2); right (10)	NA
Current marital status	Never married (11); married (5); divorced (2); domestic partnership (1)	Never married (8); separated (1); married (3); divorced (1); domestic partnership (1)	Never married (11); separated (1); married (4); divorced (2); domestic partnership (4); widowed (1)	Never married (6); separated (1); married (4); divorced (1)	NA

MADRS, Montgomery–Åsberg Depression Rating Scale; MD, major depressive disorder; CAM, Centre for Addiction and Mental Health; MCU, McMaster University; UBC, University of British Columbia; TGH, Toronto General Hospital; QNS, Queen's University; UCA, University of Calgary, NA, not applicable.

a Participants were asked which of the presented race/ethnicity categories they most closely identified with. Categories were based on Canadian census questionnaires (Statistics Canada, 2006).

We completed power analyses prior to our planned analyses, using G*Power 3.1 (Faul et al., 2007). We estimated the effect size for the comparison of the common and individual effects based on results from Gratton et al. (2018) and found that our sample provides high statistical power (>0.99) for such effects. For effects that have not been investigated previously (e.g., sex, MD diagnosis, and treatment response), we calculated statistical power for canonical small (Cohen's *d *= 0.2), medium (*d *= 0.5), and large (*d *= 0.8) effect sizes, which revealed we had sufficient power to detect large and medium effects (power >0.86). This was sufficient for our study as we were interested in determining which sources of variance contributed the most and were less interested in small effects.

### Tasks

During each scanning session, participants completed a 10 min eyes-open resting-state scan. They also performed a 10 min affective go/no-go task, where they saw squares and circles on top of irrelevant emotional images (faces with angry or neutral expressions). They were instructed to press a button every time a circle appeared and inhibit their response when they saw a square. Stimuli were presented in a mixed block-event series design, with each block either showing only angry or only neutral faces. Participants completed 16 blocks in the same order. Lastly, they performed an 11.5 min Monetary Incentive Delay (or anhedonia) task, during which they were instructed to press a button while a red square was presented on the screen. Before each target, a cue appeared indicating whether or not a reward would be given for a successful trial. Participants received feedback on each trial regarding response accuracy and reward status. These tasks have been previously described in Lam et al. (2016) and MacQueen et al. (2019). As we were interested in brain activity unrelated to the task, no data were excluded based on performance.

### fMRI data collection

Data were collected at the six sites using four different models of MRI scanners. All were 3.0 tesla systems with multicoil phased-array head coils. Extensive quality control and standardization procedures were employed to ensure that data could validly be aggregated across scanner types and recording sites (Glover et al., 2012; MacQueen et al., 2019). The influence of variation across scanners on our analyses was also explored through supplementary analyses (see below, Supplementary analyses). Participants were scanned three times, at baseline, Week 2, and Week 8 of the study. Whole-brain T2*-sensitive blood oxygenation level-dependent (BOLD) echo planar imaging (EPI) series was used to acquire the functional images with the following parameters: voxel dimensions (in mm), 4 × 4 × 4; echo time (TE), 25 or 30 ms; repetition time (TR), 2 s; flip angle, 75° or 90°; field of view (FOV), 256 mm; matrix, 64 × 64; number of slices, 34–40; acquisition order, interleaved (Lam et al., 2016; MacQueen et al., 2019).

10.1523/ENEURO.0286-23.2024.d1 Analysis scripts Download Analysis scripts, ZIP file.

### fMRI preprocessing

fMRI data were preprocessed with the OPPNI pipeline (Churchill et al., 2015, 2017; https://github.com/raamana/oppni) using the same parameters for resting state and task data. This pipeline involves (1) rigid-body motion correction (MOTCOR) via AFNI's 3dvolreg registering all volumes to the volume with the least amount of head displacement; (2) censoring (CENSOR) as implemented in (Campbell et al., 2013; software available at nitrc.org/projects/spikecor_fmri), which identifies whole-volume outliers based on a combination of signal change measures (i.e., the framewise displacement of motion parameter estimates and the root-mean-square change in signal intensity over all voxels) and interpolates them; (3) slice time correction (TIMECOR) using AFNI's 3dTshift with Fourier interpolation; (4) spatial smoothing across MRI scanners from different sites to the smoothness level of FWHM = 6 mm in three directions (*x*, *y*, *z*) using AFNI's 3dBlurToFWHM module; (5) application of a binary mask to exclude nonbrain voxels obtained with the 3dAutomask algorithm in AFNI using default parameters to all EPI volumes; (6) using the first part of the PHYCAA + algorithm to estimate task-, run-, and subject-specific neural tissue masks (Churchill and Strother, 2013; software available at nitrc.org/projects/phycaa_plus) and perform neuronal tissue masking; (7) concurrent regression of low frequency temporal trends, head motion estimates (principal components preserving 85% of the variance in motion parameter estimates obtained from MOTCOR), and global signal modulations using multiple linear regression (Carbonell et al., 2011; Churchill et al., 2012a,b); (8) data-driven physiological correction (PHYPLUS) using the second part of the data-driven PHYCAA+ algorithm to estimate and remove physiological noise (e.g., related to heart rate and respiration); (9) low-pass filtering (LOWPASS) at 0.10 Hz with a linear filter; and (10) spatial normalization to the structural MNI152 template (4 mm resolution; sNORM) using FSL's FMRIB's Linear Image Registration Tool. In addition, the data were directly registered to a sample-specific EPI template through an affine transformation followed by a nonlinear transformation following the EPInorm strategy (Calhoun et al., 2017).

Next, we regressed out task-evoked activity from the affective go/no-go and anhedonia task data for each participant and session, as task-evoked activity has been found to inflate functional connectivity values (Cole et al., 2019). This and the following steps were completed in MATLAB 2018b (The MathWorks). We created regressors for each relevant event type from the task (described in more detail below) and used the regress.m function in MATLAB to remove activity related to these task events from the data. As differences in the shape and magnitude of the hemodynamic response function (HRF) have been found in patients with depression compared with controls (L. Wang et al., 2008; Kinou et al., 2013; Gao et al., 2016; Husain et al., 2020), and canonical HRF task regression has been found to increase false positives in connectivity (Cole et al., 2019), we adopted a more flexible approach as suggested by Cole et al. (2019). Namely, we used the code shared by them (link: https://github.com/ColeLab/TaskFCRemoveMeanActivity/) to apply finite impulse response (FIR) task regression. Specifically, we used the convertTaskTimingToFIRDesignMat.m function, which creates a set of regressors for each type of event that is time-locked to the events but allows for a flexible fit in terms of the exact shape of the HRF for each individual, as long as it is consistent across blocks/trials. See Cole et al., for more information on this method.

For the affective go/no-go task, we used five primary regressors: one for the instruction slides and one each for the four types of blocks (angry go, angry no-go, neutral go, and neutral no-go blocks). For the anhedonia task, we used seven primary regressors: two for the cue (one signaling a reward trial, the other signaling a no reward trial), one for the target, and four for each type of feedback (hit on a reward trial, hit on a no reward trial, miss on a reward trial and miss on a no reward trial). The matrices containing these binary regressors were entered as input to the convertTaskTimingToFIRDesignMat.m function, with 10 as the firLag parameter. This is advised to be ∼20 s, which in our case is 10 TRs. The regress.m function outputs the residuals from the regression model, which were used for the analyses. This function also corrects for collinearity among regressors, which is a common issue with the type of FIR filter we use. While regression of task-evoked activity is never perfect, previous studies have found similar whole-brain functional connectivity with and without this step (Gratton et al., 2018; Elliott et al., 2019).

While most preprocessing steps completed as part of the OPPNI pipeline corresponded well to the preprocessing steps used by Gratton et al. (2018), there was one step missing. Namely, Gratton et al. demeaned their data prior to calculating connectivity. Therefore, we also applied this step to our data by subtracting the mean of the time course for each voxel from each data point for that voxel. In addition, it may be of note that we performed the regression of task effects after smoothing on surface weighted data, while Gratton et al. performed smoothing on volume data after the regression of task effects.

According to Cho et al. (2021), concatenating functional resting state and task data first and then calculating FC versus first calculating FC and then averaging over resting state and task data result in highly similar results for data segments of lengths 5, 10, and 15 min. However, for full (compared with partial) correlations, the first strategy yielded somewhat higher reliability of FC (Cho et al., 2021). Therefore, for our study we chose to first concatenate the three task scans (resting state, affective go/no-go, and anhedonia) from each recording session and then calculate FC on the concatenated data for each individual and recording session. This way we had time courses of ∼30 min per participant and session.

### Parcellation

We parcellated each individual's brain into 333 regions based on the atlas created by Gordon et al. (2016). They used a resting-state functional connectivity (RSFC) boundary mapping technique and showed that their solution performed better compared with previous area-level parcellation models, especially for use in individual participants. More specifically, we used the MNI template (2 × 2 × 2 mm) as shared by Gordon et al. (http://www.nil.wustl.edu/labs/petersen/Resources.html) and matched it to the spatial resolution and space of our fMRI data (4 × 4 × 4 mm) using FSL. We first registered a standard MNI brain to the EPI template our functional data were registered to and then applied the same transformation to the parcellation file using the flirt function. To make sure the region labels were maintained, we used nearest neighbor interpolation for the second transformation. The code for these transformations is saved in MNI2EPI_code.txt. Seven regions (six in the orbitofrontal cortex and one in the right inferior temporal gyrus) did not have coverage and were therefore excluded. The average time course over the voxels within each region was extracted and used for the functional connectivity estimation.

### Data analysis

**Figure 1.**
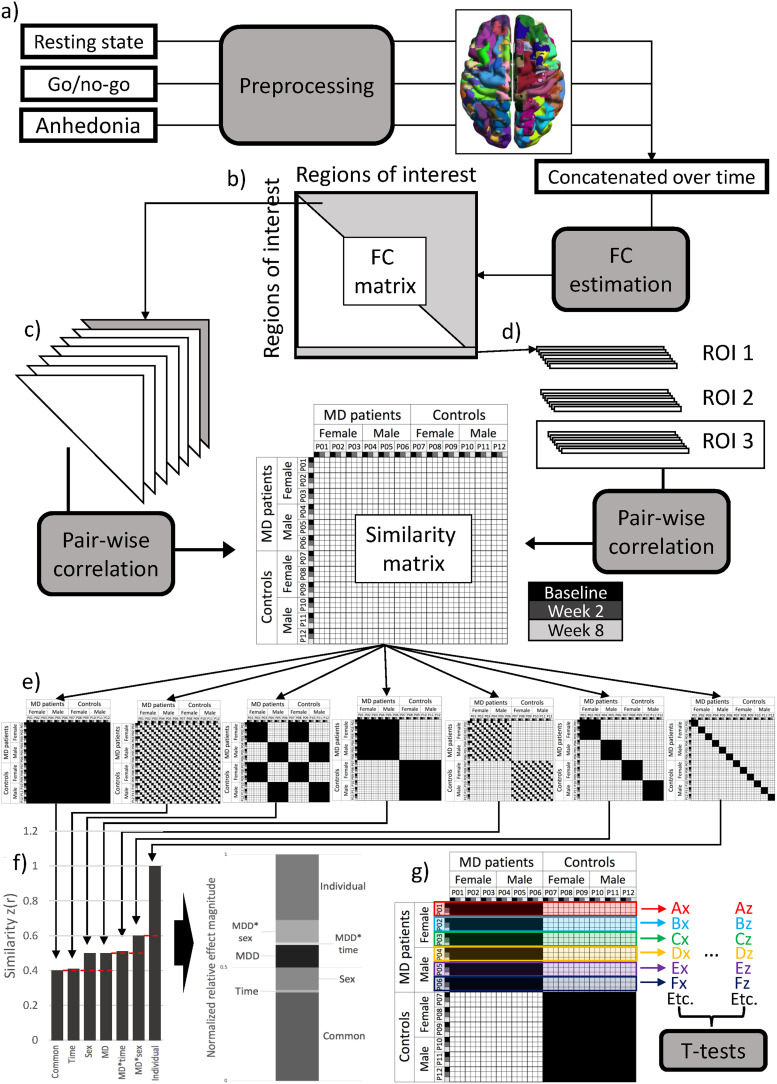
Overview of analysis procedure. The analysis for patients and controls is used as an example, but steps were the same for analyses with controls only and patients only. ***a***, The fMRI data from resting state, affective go/no-go, and anhedonia (or monetary incentive delay) tasks were preprocessed using the OPPNI pipeline (Churchill et al., 2015, 2017), parcellated into 326 regions and concatenated for each participant and session. ***b***, FC was calculated as the correlation between the time courses of each region pair, resulting in a region-by-region FC matrix. ***c***, For the whole-brain analyses, the upper triangle (gray) of the FC matrix from each participant and session was correlated with that of every other participant and session to create a similarity matrix. ***d***, For the region-specific analyses, a similarity matrix was constructed for each ROI by correlating each ROI's row in the correlation matrix with that of the same row of other participants and sessions. ***e***, The contribution of each source of variance was estimated by calculating the average similarity over different configurations of the similarity matrix. From left to right, the presented patterns display the configuration for the calculation of the common effect (similar FC across all participants and sessions), the session effect (similar FC across participants within sessions), the sex effect (similar FC among female and among male participants), the MD effect (similar FC among patients with an MD diagnosis and among controls), the MD × session interaction (similar FC among patients and controls within sessions), the MD × sex interaction (similar FC among female patients, male patients, female controls, and male controls), and the individual effect (similar FC within individuals across sessions). ***f***, From the average similarity for each effect, we calculated the normalized relative effect magnitude by first subtracting the baseline for each effect (indicated by the dashed red lines) and then dividing by the total relative similarity. For the region-specific analyses, we visualized the normalized relative effect magnitudes but did not perform statistical comparisons. ***g***, For the whole-brain analyses, we also calculated the average similarity for each effect per individual by taking the average of the patterns for the three rows representing each participant. The example shows this for the MD effect. For each participant, indicated by different color outlines, we calculated the average similarity of that participant to all other participants and sessions within the same group (i.e., by taking the average across black squares within the three rows representing the three recording sessions of that participant). This way, we calculated each of the effects shown in part ***e*** of the figure at an individual level as well. These participant-specific average similarity values were then entered into dependent samples *t* tests to examine differences in effect magnitudes. Each capital letters (***A–F***) represent individuals and the lowercase letters (*x*–*z*) the different effects (i.e., Ax is the MD effect magnitude for participant 1, Az is the effect magnitude for a different effect for that same participant). FC, functional connectivity; ROI, region of interest; MD, major depressive disorder.

We performed analyses on controls-only, patients and controls together, and patients-only to examine the contribution of different sources of variance following the methodology in Gratton et al. (2018). First, we estimated functional connectivity between all regions within each individual and session using product-moment correlations between the ROI time courses and applied a Fisher's *z*-transformation. Next, we correlated the upper half of each session and individual's whole-brain connectivity matrix with that of each other session (including sessions from the same individual) and individual's whole-brain connectivity pattern to construct a similarity matrix (Fig. 1*c*). In this similarity matrix, each row and column represented a specific individual and session, together depicting the similarity of whole-brain functional connectivity for different pairs of individuals and sessions. We applied Fisher's *z*-transformation to these similarity matrices as well.

To quantify the magnitude of different sources of variance, we calculated the average over different parts of the similarity matrix (as illustrated in Fig. 1*e*). The diagonal of the matrix (all ones by definition) was not included in the averages. Each average was also calculated within the three rows representing each participant to get an estimate of each effect based on the similarity values of that participant with their own and the other participants’ data (Fig. 1*g*). These participant-specific values were submitted to dependent sample *t* tests in MATLAB with permutation testing (1,000 permutations) to estimate significance. Specifically, for each comparison, the data were randomly shuffled between the two effects within an individual for each permutation. While the data were not fully independent, they were exchangeable, which is what is required for permutation testing (LaFleur and Greevy, 2009).

We compared each effect with its baseline (Fig. 1*f*) and the other effects of the same type (e.g., main effects to other main effects), meaning we conducted four statistical comparisons for the controls-only and 10 comparisons for each of the patients and controls and patients-only samples. We used the false discovery rate (FDR) method to correct for multiple comparisons (Benjamini and Yekutieli, 2001). We calculated the normalized relative effect magnitude as an indication of effect size. First, we subtracted the baseline from each effect (Fig. 1*f*) and then divided by the sum of all baseline-corrected magnitudes. As such, normalized relative effect magnitude values reflect the proportion of each effect that is unique, i.e., not explained by its baseline, relative to the combined unique magnitude of all effects. For the interaction effects, we used the main effect with the highest similarity as baseline (e.g., if the MD effect was larger than the sex effect, the MD effect would serve as the baseline for the MD × sex interaction). If an effect had a lower magnitude than its baseline, the baseline-corrected magnitude was set to zero.

We also examined the distribution of these effects in the brain by conducting the same analysis for each region separately; i.e., instead of correlating the upper triangle of the whole-brain connectivity matrix, we correlated the connectivity estimates of one brain region with all other brain regions across participants and sessions to create a similarity matrix for each region (Fig. 1*d*). We calculated the normalized relative effect magnitude for each effect and region to illustrate where in the brain the effects were most prominent but did not perform statistical analyses on these region-specific estimates. See the preregistration document for a detailed description of all data analysis steps (https://osf.io/79gv8/?view_only=9e105962ce4c4ebf8cf35393471a7b69).

### Supplementary analyses

We conducted several supplementary analyses to ensure the validity of our main analyses. First, we performed the whole-brain analyses excluding ROIs with fewer than eight voxels (45/326 ROIs excluded), as the average time courses extracted from these ROIs may have been less reliable/accurate. Next, we conducted the whole-brain analyses for patients and controls and patients only on scanner, sex, age, and education matched subsamples to examine if these factors may have influenced our results. In addition, these analyses served to ascertain if unequal group sizes may have distorted our findings. We matched participants to the smallest subgroup in each analysis, creating a subsample of 72 and 48 participants for the patients and controls and patients-only analyses, respectively. Two (female vs male) by 2 (controls vs patients or responders vs nonresponders) ANOVAs revealed no statistically significant differences between groups in age and years of education (all *F*s < 1.67; all *p*s > 0.20). The number of female and male participants was equal in the matched groups, and the distributions of scanner types varied little across groups (max. difference was 4 participants).

To examine the potential impact of censoring images in our data, we conducted a series of ANCOVAs with the number of censored frames per participant as a covariate for the comparisons between effects for the controls only, patients and controls, and patients-only samples. Lastly, we examined the reliability of our connectivity data by splitting each individual's data in half and correlating connectivity calculated from one half of the data with connectivity calculated from increasing amounts from the other half of the data (from ∼5 to ∼45 min). For both the split-half and the shorter amounts, we epoched data from each task and session into ∼2.5 min sections and pseudorandomly selected epochs from different tasks and sessions for each segment (i.e., all segments included both resting state and task data and sections from the first and second half of the session recordings). We conducted this reliability analysis on data from control participants only, because we expected connectivity for participants with MD to change over time due to treatment effects.

### Code accessibility

All custom codes for these analyses are available at https://osf.io/79gv8/files/github?view_only=9e105962ce4c4ebf8cf35393471a7b69. Any functions used but not provided are publicly available, either as built-in MATLAB functions or from the EEGLAB Toolbox (version 14.1.2.0; Delorme and Makeig, 2004). The code was run on an Apple Desktop with a MacOS operating system (High Sierra). The data used in this manuscript have been collected as part of the CAN-BIND initiative, an Integrated Discovery Program of the Ontario Brain Institute (OBI). OBI has released data from CAN-BIND's foundational study, which aims to identify biomarkers that predict treatment response in people with depression. The dataset currently available on Brain-CODE includes baseline and longitudinal data from participant follow-up visits in Weeks 2–8 of the study (Phase 1). All data have been standardized, cleaned, and curated to maximize utility for analysis across different data modalities, and imaging data were converted to a BIDS-friendly naming convention. For access requirements, please visit OBI's Brain-CODE Neuroinformatics Platform (https://www.braincode.ca/) or email info@braininstitute.ca.

## Results

### Participants

The demographic information for patients and controls, broken into female and male participant subgroups, is presented in Table 1. Two (patients vs controls) × 2 (female vs male participants) ANOVAs indicated there was no significant effect of age (all *F*s < 1.48; all *p*s > 0.23). However, there was a significant main effect of MD diagnosis for years of education (*F*_(1,103)_ = 11.34; *p* = 0.001). Namely, participants in the control group (*M *= 18.49; SD = 1.97) had a higher number of years of education compared with the participants in the patient group (*M *= 17.15; SD = 1.79), which is consistent with previous studies (Elderkinthompson et al., 2003; Selvi et al., 2010; Salmela et al., 2021). We examined the potential impact of this difference in our supplementary analyses and found no evidence of an effect. Table 2 displays the clinical and demographic information for responder and nonresponder groups, again broken down into female and male participant subgroups. As expected, there was a significant main effect of response for MADRS symptom scores at Week 2 (*F*_(1,64)_ = 11.61; *p *= 0.001) and Week 8 (*F*_(1,64)_ = 119.95; *p *< 0.001). Apart from that, no significant effects were observed.

### Sources of variance in whole-brain FC

**Figure 2.**
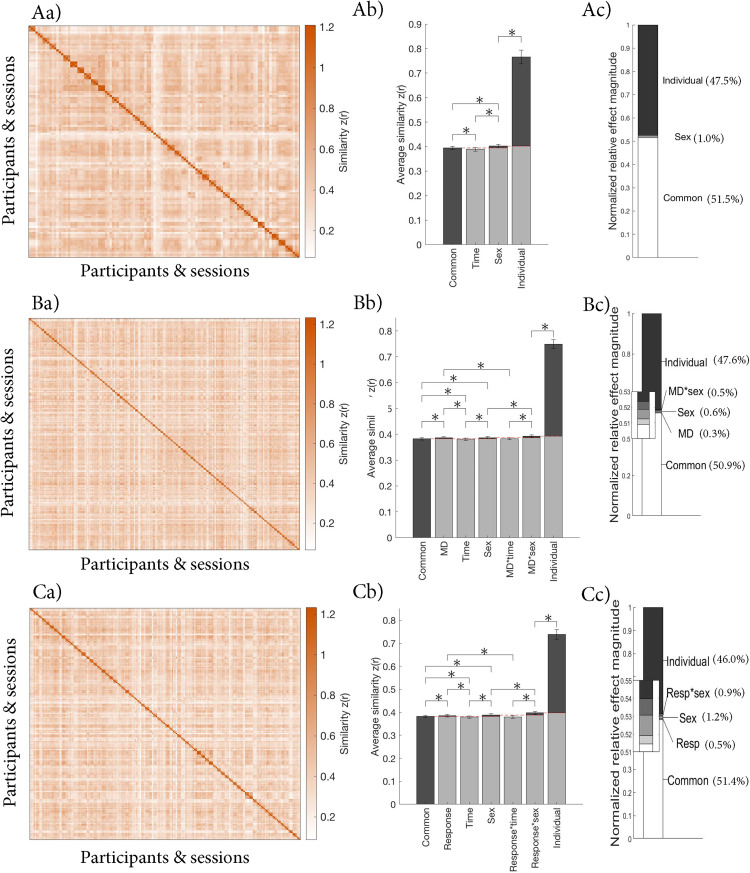
Contributions of different sources of variance in FC in ***A***, controls only, ***B***, patients and controls, and ***C***, patients only. ***a***, Similarity matrix displaying the Fisher transformed correlations between the whole-brain FC of different participants and sessions. The matrices are organized by group (sex and MD diagnosis or response), individual, and session (baseline, Week 2, and Week 8) as illustrated in Figure 1. ***b***, Average similarity for each effect. Figure 1*e* illustrates how each average was calculated. The red dashed lines indicate the baseline for each effect, which was subtracted to calculate the normalized relative effect magnitudes. ***c***, Normalized relative effect magnitude for each effect.

We investigated the contributions of different sources of variance relative to their baselines in controls only, patients and controls together, and patients only. The pattern was similar across all three samples (Fig. 2 and Table 3). Namely, common FC across all participants and conditions (common effect) and individual-specific FC (individual effect) contributed most to the observed variance (normalized relative effect magnitude = 50.9–51.5% and 46.0–47.5%, respectively). The individual effect was significantly bigger than its baseline in all samples (*p*s < 0.001). The contributions of sex, MD, response effects, and their interactions were significant but small (normalized relative effect magnitude = 0.3–1.2%; *p*s < 0.001). The session effect and its interactions were significantly smaller than their baselines (*p*s < 0.001).

**Table 3.** Results of paired samples *t* tests to compare magnitude of effects in controls only, patients and controls, and patients only

Sample	Comparison	*t*-value	DF	SD	Uncorrected *p*-value	Cohen's *d*
Controls only	Common > session	9.32	38	0.004	<0.001*	−1.49
	Common < sex	−7.85	38	0.006	<0.001*	1.26
	Session < sex	−10.48	38	0.008	<0.001*	1.68
	Sex < individual	−15.27	38	0.149	<0.001*	2.44
Patients and controls	Common < MD	−6.52	106	0.006	<0.001*	0.63
	Common > session	6.72	106	0.002	<0.001*	−0.65
	Common < sex	−7.35	106	0.006	<0.001*	0.71
	MD > session	8.20	106	0.007	<0.001*	−0.79
	MD = sex	−0.62	106	0.009	0.530	0.06
	Session < sex	−8.69	106	0.007	<0.001*	0.84
	MD > MD × session interaction	11.89	106	0.003	<0.001*	−1.15
	Sex < MD × sex interaction	−7.44	106	0.008	<0.001*	0.72
	MD × session interaction < MD × sex interaction	−12.11	106	0.009	<0.001*	1.17
	MD × sex interaction < individual	−25.40	106	0.145	<0.001*	2.46
Patients only	Common < Response	−3.95	67	0.01	<0.001*	0.48
	Common > session	9.72	67	0.00	<0.001*	−1.18
	Common < sex	−4.88	67	0.01	<0.001*	0.59
	Response > session	6.68	67	0.01	<0.001*	−0.81
	Response = sex	−1.41	67	0.01	0.179	0.17
	Session < sex	−6.41	67	0.01	<0.001*	0.78
	Response > response × session interaction	13.09	67	0.00	<0.001*	−1.59
	Sex < response × sex interaction	−4.71	67	0.02	<0.001*	0.57
	Response × session interaction < response × sex interaction	−7.45	67	0.02	<0.001*	0.90
	Response × sex interaction < individual	−20.18	67	0.14	<0.001*	2.45

* Significant effects after FDR correction.

**Figure 3.**
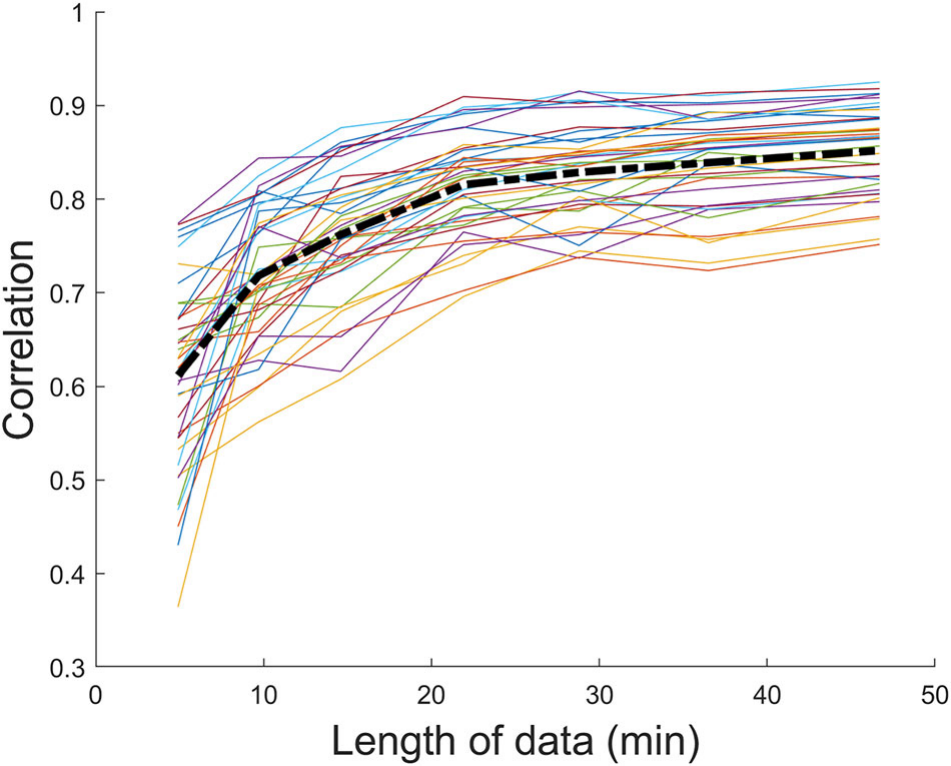
Correlation between parcel-level connectivity calculated using split-half sampling of data for control participants, where half of the data (∼45 min) was compared with increasingly longer segments of data from the other half. Colored lines represent individual participants, while the black dotted line represents the average across participants.

The supplementary analyses excluding ROIs with fewer than 8 voxels yielded highly similar results as reported in the main analyses. Namely, the main contributors to the overall variance in all three samples were the common and individual effects, while sex, MDD diagnosis, response to treatment, and their interactions contributed a small but significant amount. The matched subsample analyses revealed the same pattern of results. This indicates that the findings above were not meaningfully influenced by differences in scanner types, years of education, or unequal group sizes. The series of ANCOVAs with the number of censored frames included as covariate showed that our results did not change apart from the difference between the sex effect and the sex × response interaction going from significant (*p* < 0.001) to marginally significant (*p* = 0.062) in the patients-only sample. The reliability analyses showed that the correlation between connectivity calculated from one half of the data (∼45 min) and shorter segments increased with segment length and reached on average ∼0.85 for the longest segment (∼45 min; Fig. 3).

### Localization of effects

**Figure 4.**
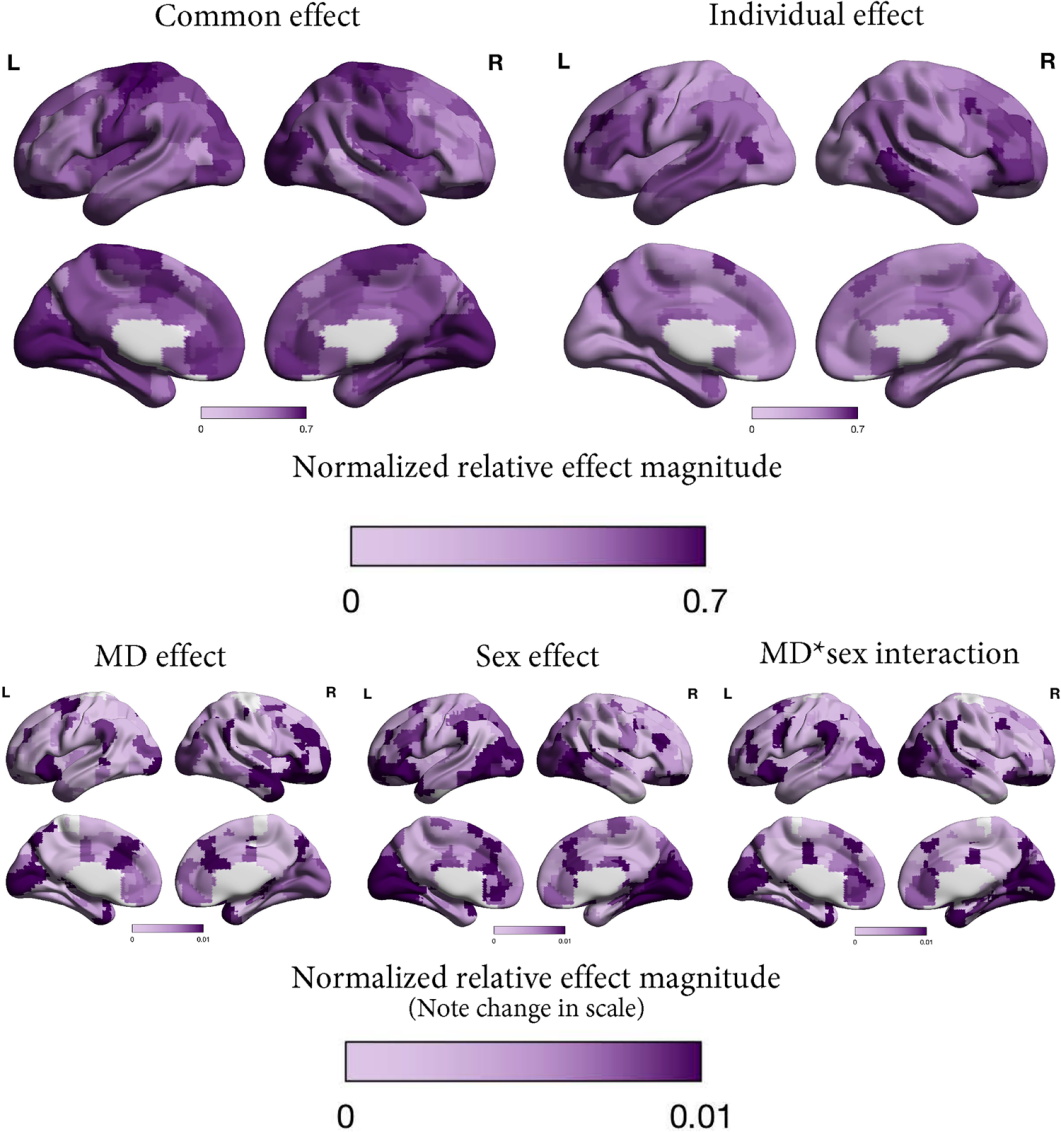
Normalized relative effect magnitude in each region of the brain for each effect (common, MD, sex, MD × sex, and individual) in patients and controls. The MD, sex, and MD × sex effects are presented on a narrower color scale so the localization of these effects can be distinguished.

**Figure 5.**
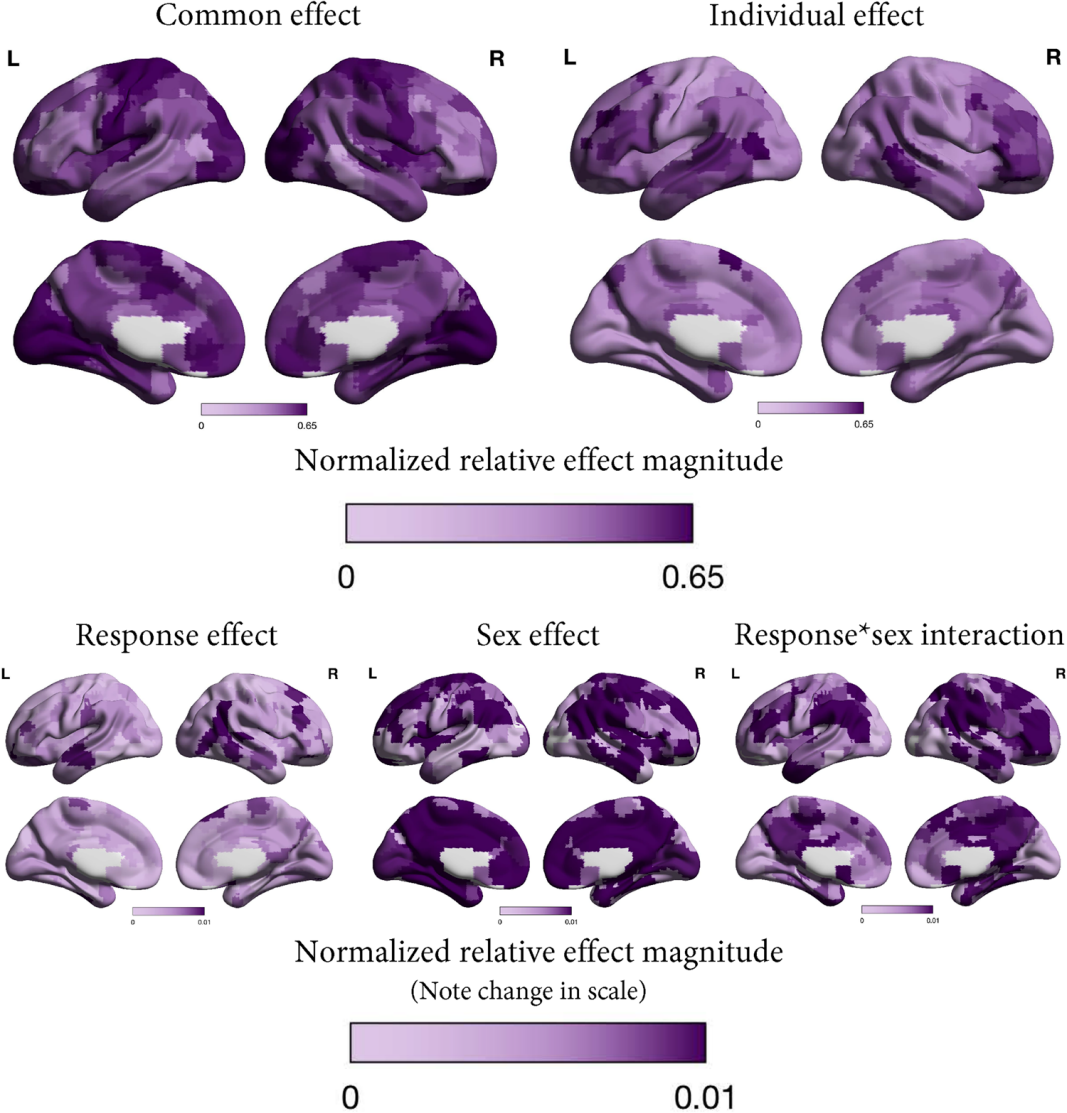
Normalized relative effect magnitude in each region of the brain for each effect (common, response, sex, response × sex, and individual) in patients only. The response, sex, and response × sex effects are presented on a narrower color scale so the localization of these effects can be distinguished.

We also illustrate the normalized relative effect magnitudes calculated per ROI to indicate where each effect was most prominently present in the brain (Figs. 4, 5). In both samples, the common effect was most strongly present in default mode network, somatosensory, motor, visual, and auditory areas. The individual effect was expressed most strongly in frontoparietal, dorsal attention, and cinguloparietal network areas. The sex, MD, and response effects and their interactions contributed relatively little, therefore the distribution of these effects cannot be seen on the same scale as the common and individual effects, so we illustrate them on a narrower scale.

### Exploratory analyses

**Figure 6.**
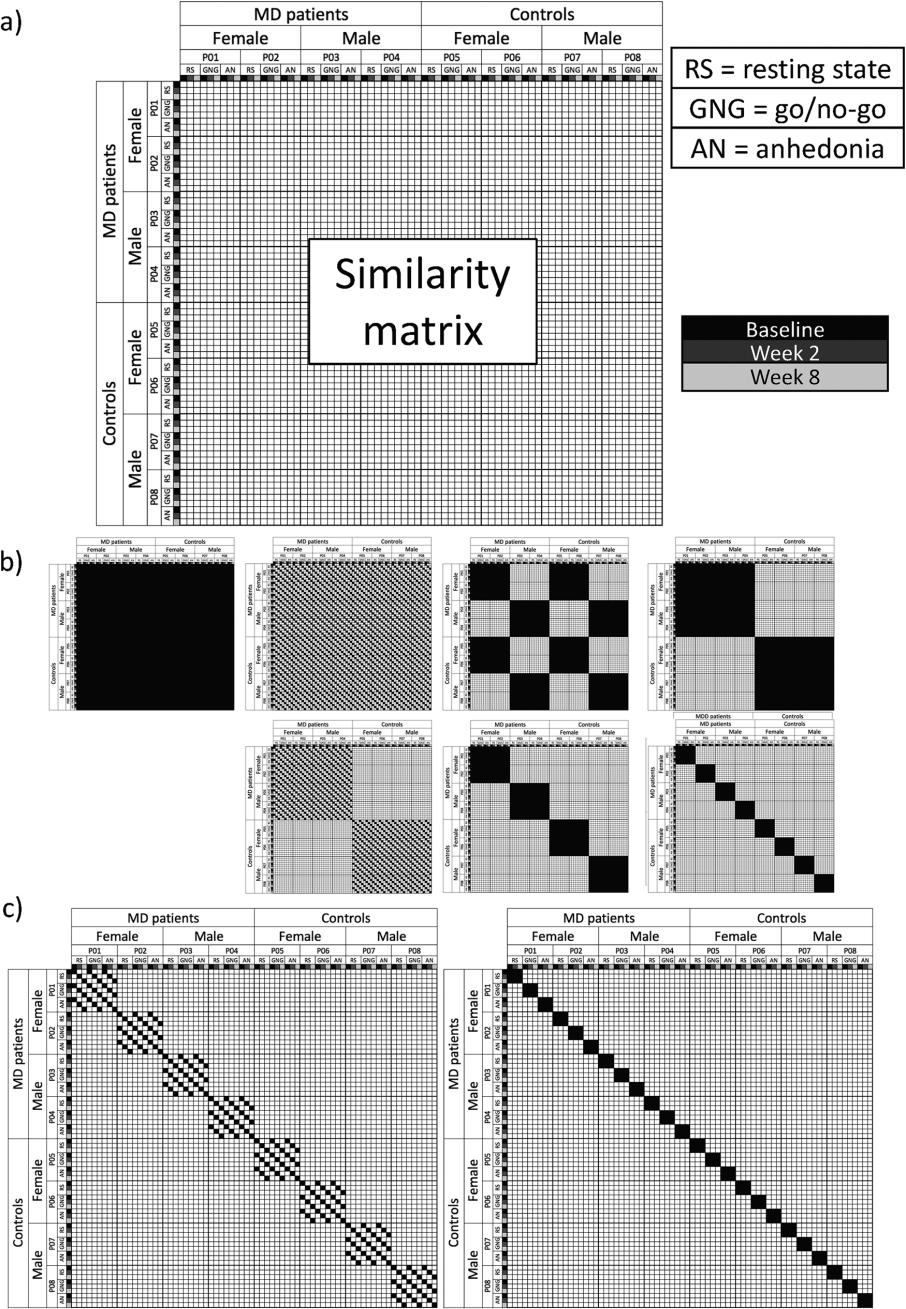
Illustrations of the similarity matrix and matrix configurations used to calculate the averages for each effect in the exploratory analyses, taking the patients and controls sample as the example. ***a***, Structure of the similarity matrix exemplified for eight participants. There is now a separate row for each participant, session, and task (instead of just for each participant and session in the main analyses). ***b***, Matrix configuration for each of the effects that were also examined in the main analyses (Fig. 1*e*). The effects were calculated by averaging over the black squares in the matrix. From left to right and top to bottom, the patterns represent the common effect (similar FC across all participants and sessions), the session effect (similar FC across participants within sessions), the sex effect (similar FC among female and among male participants), the MD effect (similar FC among patients with an MD diagnosis and among controls), the MD × session interaction (similar FC among patients and controls within sessions), the MD × sex interaction (similar FC among female patients, male patients, female controls, and male controls), and the individual effect (similar FC within individuals across sessions). ***c***, Matrix configurations of the effects only examined in the exploratory analyses. The individual × time interaction (similar FC within individual and session) is illustrated on the left, while the individual × task interaction (similar FC within individual and task) is illustrated on the right.

As the results above point to a large contribution of individual variation relative to other sources of variance (e.g., MD diagnosis, sex, and response to treatment), we further explored the contributions of individual × task and individual × session interactions (Fig. 6) in exploratory analyses. Instead of pooling data across tasks, we calculated functional connectivity for each task and session. We had 9.8 min of data for the resting state and affective go/no-go task, and 11.5 min for the anhedonia task, which provide ∼72–82% reliability for the individual connectivity matrices (Laumann et al., 2015; Gordon et al., 2017).

**Figure 7.**
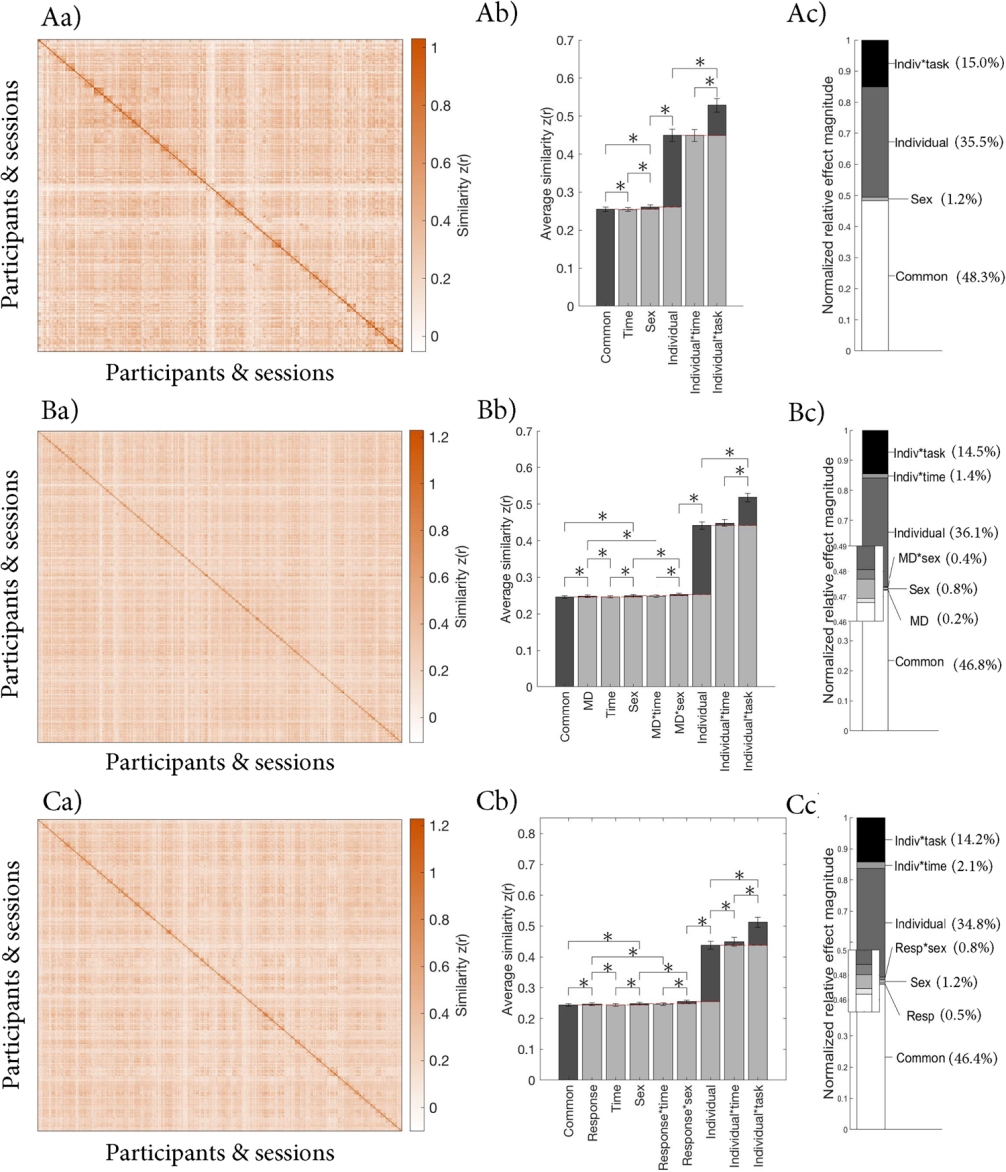
Contributions of different sources of variance in FC as examined in our exploratory analyses in ***A***, controls only, ***B***, patients and controls, and ***C***, patients only. ***a***, Similarity matrix displaying the Fisher transformed correlations between the whole-brain FC of different participants and sessions. The matrices are organized by group (sex and MD diagnosis or response), individual, task (resting state, affective go/no-go, and anhedonia), and session (baseline, Week 2, and Week 8) as illustrated in Figure 6. ***b***, Average similarity for each effect. See Figure 6 for the pattern across which each average was calculated. The red dashed lines indicate the baseline for each effect, which were subtracted to calculate the normalized relative effect magnitudes. ***c***, Normalized relative effect magnitude for each effect.

These exploratory analyses revealed a similar pattern of results for the effects examined with the pooled data (Table 4 and Fig. 7). The only notable difference was that the session effect was no longer smaller than its baseline for controls and patients and for patients-only. In addition, we found a significant contribution of the individual × task interaction for all three samples (normalized relative effect magnitude = 14.2–15.0%; all *p*s < 0.001). Interestingly, the individual × session interaction was significant in patients only (normalized relative effect magnitude = 2.1%; *p *= 0.016), but not in the other two samples.

**Table 4.** Results of paired samples *t* tests to compare magnitude of effects in our exploratory analyses in controls only, patients and controls, and patients only

Sample	Comparison	*t*-value	DF	SD	Uncorrected *p*-value	Cohen's *d*
Controls only	Common > session	6.75	38	0.001	<0.001*	−1.08
	Common < sex	−5.95	38	0.006	<0.001*	0.95
	Session < sex	−6.67	38	0.006	<0.001*	1.07
	Sex < individual	−16.66	38	0.071	<0.001*	2.67
	Individual = Individual × session interaction	0.02	38	0.033	0.98	0.00
	Individual < Individual × task interaction	−12.46	38	0.040	<0.001*	1.99
	Individual × session interaction < Individual × task interaction	−7.22	38	0.069	<0.001*	1.16
Patients and controls	Common < MD	−5.54	106	0.004	<0.001*	0.54
	Common = session	0.01	106	0.001	0.99	0.00
	Common < sex	−7.55	106	0.004	<0.001*	0.73
	MD > session	5.38	106	0.004	<0.001*	−0.52
	MD = sex	−1.80	106	0.006	0.072	0.17
	Session < sex	−7.24	106	0.005	<0.001*	0.70
	MD > MD × session interaction	4.16	106	0.001	<0.001*	−0.40
	Sex < MD × sex interaction	−6.58	106	0.006	<0.001*	0.64
	MD × session interaction < MD × sex interaction	−9.39	106	0.006	<0.001*	0.91
	MD × sex interaction < individual	−25.64	106	0.076	<0.001*	2.48
	Individual = Individual × session interaction	−2.01	106	0.037	0.051	0.19
	Individual < Individual × task interaction	−15.76	106	0.050	<0.001*	1.52
	Individual × session interaction < Individual × task interaction	−8.76	106	0.082	<0.001*	0.85
Patients only	Common < Response	−6.04	67	0.004	<0.001*	0.73
	Common = session	0.64	67	0.001	0.53	−0.08
	Common < sex	−5.43	67	0.006	<0.001*	0.66
	Response > session	6.05	67	0.004	<0.001*	−0.73
	Response = sex	−1.90	67	0.007	0.058	0.23
	Session < sex	−5.28	67	0.007	<0.001*	0.64
	Response > Response × session interaction	3.73	67	0.001	0.002*	−0.45
	Sex < Response × sex interaction	−4.18	67	0.012	0.001*	0.51
	Response × session interaction < Response × sex interaction	−5.08	67	0.014	<0.001*	0.62
	Response × sex interaction < individual	−19.07	67	0.079	<0.001*	2.31
	Individual < Individual × session interaction	−2.43	67	0.038	0.016*	0.29
	Individual < Individual × task interaction	−11.12	67	0.056	<0.001*	1.35
	Individual × session interaction < Individual × task interaction	−5.92	67	0.089	<0.001*	0.72

* Significant effects after FDR correction.

**Figure 8.**
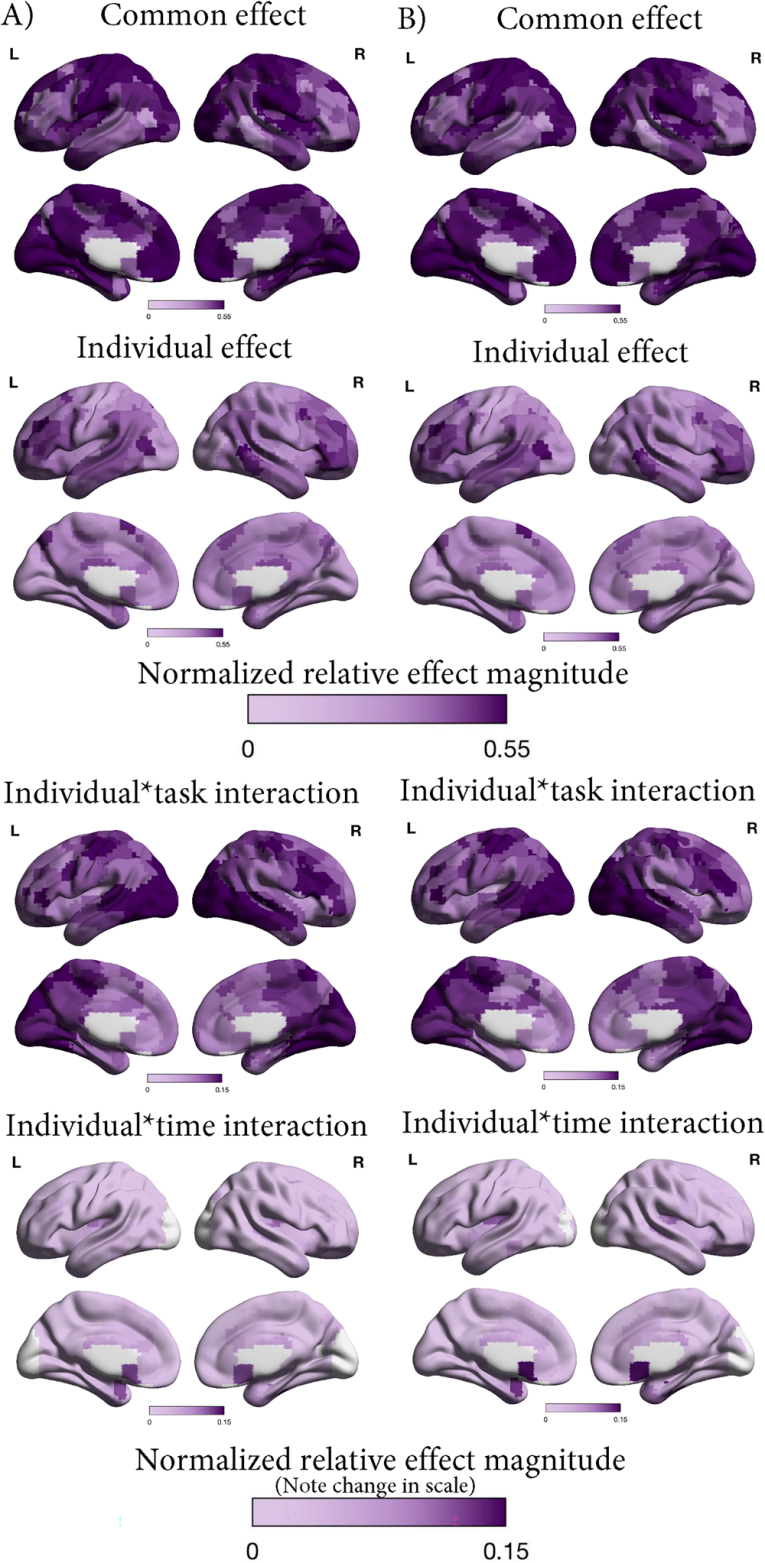
Normalized relative effect magnitude in each region of the brain for selected effects from the exploratory analyses (common, individual, individual × task, and individual × session) in ***A***, patients and controls and ***B***, patients only.

We also examined regional localization for these effects (Fig. 8). The individual × task interaction was most prominent in the right lateral prefrontal cortex, bilateral visual areas, and left somatosensory areas. The individual × session interaction was expressed primarily in bilateral subgenual cingulate cortex, medial temporal lobes, and temporal poles.

## Discussion

In this study, we investigated the relative contributions of several sources of variance observed in FC data collected across 8 weeks from participants with MD and controls, with a particular interest in the relative contribution of clinical characteristics including MD diagnosis and response to treatment. While commonly studied sources (MD diagnosis, response to/change with treatment, and biological sex) did contribute to the overall variance, these effects were small (0.3–1.2%) relative to common and individual-specific FC, which explained >95% of the variance in the data across analyses. Supplementary analyses showed that ROI size, scanner types, demographics, or unequal group sizes did not meaningfully influence our findings. The localization analyses showed brain areas with low and high individual variation in line with previous findings in neurotypical individuals.

The pattern of relative contributions of common versus individual variance in FC in our study resembled that reported by Gratton et al. (2018) in a small (*N* = 10) highly sampled group of neurotypical individuals (i.e., that common connectivity across all sessions and participants and individual differences contributed most to the overall variance). The regions where individual variation was low (strong common FC) or high (strong individual-specific FC) further matched previous work with neurotypical adults (Mueller et al., 2013; Chen et al., 2015; Gratton et al., 2018; Kong et al., 2019; Seitzman et al., 2019). Namely, individual variation was low in primary sensory and motor, as well as some default mode network areas, and high in lateral (pre)frontal areas and midtemporal areas. Whereas sensorimotor networks develop early in life and are therefore more strongly guided by genetic determination (but also see Graff et al., 2022), cognitive control and attention networks continue to develop long after birth and may therefore be more impacted by environmental factors, leading to greater interindividual variation (Mueller et al., 2013). These findings indicate a degree of stability in the distribution and magnitude of shared and individual variation in FC regardless of mental health and treatment status.

To our knowledge, this study is the first to examine the relative magnitudes of effects related to MD diagnosis, response to treatment, and biological sex. The results suggest that these effects were small, which is consistent with studies revealing significant but small group differences next to a great degree of overlap in structural and functional brain features across groups (Joel et al., 2015; Winter et al., 2022). Considered together with large commonalities and individual differences, the small relative magnitude of these group effects may contribute significantly to the variable findings in neuroimaging features of MD and treatment response to date. Further, these findings may also shed some light on why, despite hopes to the contrary, it is proving difficult to use such features to predict treatment success at an individual level (Etkin, 2019; Gratton et al., 2020). As explained by Arbabshirani et al. (2017), large overlap between groups generally results in low classification rates, even when mean group differences are highly significant. Next to discriminatory value, ensuring that individual estimates are reliable (e.g., through the collection of greater amounts of data per individual) will likely be essential for neuroimaging findings to aid in predicting individual treatment outcomes (Gratton et al., 2020). Approaching this complex issue through multiple pathways that focus on both group differences and individual variation may be required for further progress in identifying predictors of treatment success that can be applied in clinical settings.

One potential exciting direction highlighted by our study is exploring the clinical utility of the substantial individual differences found here. While most variation was not related to group membership based on MD diagnosis or treatment response, individual variation could still be relevant in the context of MD and its treatment. Particularly the high individual variation in regions involved in cognitive control may be important, as MD has been proposed to involve abnormal functional connectivity within and among the CCN and other resting state networks (DMN and SN; Mulders et al., 2015; Brakowski et al., 2017). As both MD symptom profiles and cognitive control processes are complex and heterogenous (Fried and Nesse, 2015; Joormann and Tanovic, 2015), a more detailed exploration of such specific features in relation to individual differences in the brain may provide more insight. Neuroimaging is particularly suited to this considering the high dimensionality and intrinsic variability of this type of data. Indeed, a recent preprint examining individual differences in brain network structure in persons with depression highlights the promise of this approach (Lynch et al., 2023). Using intensive longitudinal neuroimaging data, they found that individual features of the salience network were related to the presence and later development of MD, as well as fluctuations in symptoms over time, pointing toward the potential clinical utility of such individual features when examined in depth.

This approach may be useful more broadly as well, as a concern raised in neuroimaging research on mental health conditions proposes that diagnostic categories may be too broad to capture specific neurobiological processes (Insel et al., 2010; Holtzheimer and Mayberg, 2011; Cuthbert, 2014; Blackburn, 2019). A related issue is that features of the brain have been found to differ depending on the stage of the mental health condition (e.g., first episode or recurrent depression; Frodl et al., 2003; Sheline et al., 2003; McGorry et al., 2006; Kronmüller et al., 2009; Yüksel et al., 2018). Several papers have therefore called for studies examining the brain in relation to more specific symptoms and/or stages of MD (McGorry et al., 2006; Holtzheimer and Mayberg, 2011; Cuthbert, 2014). Investigating the clinical relevance of individual variation in the brain using such a fine-grained approach may help disentangle if and how individual differences relate to depression symptoms and treatment effects.

Contrary to our hypotheses, the similarity of FC across participants within time points (i.e., the effect of consistent change over time across participants) was smaller than common FC in all main analyses. We believe that this happened because, in the design of the similarity matrix for this study, time-specific FC did not include any similarity values calculated within an individual (Fig. 1*e*). Since similarity within individuals over time points was high, the fact that such similarities were not part of the average for the time-specific FC may have led to lower averages compared with the common FC similarity, which did include similarity within individuals. This is supported by the observation that the similarity estimates for time-specific FC were no longer significantly smaller in two of the exploratory analyses, where within individual similarity values were part of the average (Fig. 6).

Even so, session-specific FC across participants did not contribute to the overall variance observed in the data beyond common FC, indicating changes over session did not occur the same way across participants, not even in patients receiving the same treatment. This finding is discrepant with previous research summarized in a systematic review that did identify similar changes across treatment (Gudayol-Ferré et al., 2015). Next to other methodological differences (e.g., whole-brain FC vs specific edges), this may be accounted for by the fact that within-subject designs studying changes over time typically calculate change within an individual and then examine consistencies in these changes across participants. Here instead, our main analyses examined similarities over time points across participants directly. Previous study designs more closely resemble our exploratory analyses, which did highlight a contribution of individual- and session-specific FC in patients exclusively, indicating an effect of antidepressant treatment over time. The localization of this effect (temporal poles, subgenual cingulate cortex, and medial temporal areas) also partly overlapped with previous findings (Gudayol-Ferré et al., 2015; Mulders et al., 2015). Notably, our approach did not require changes to be in the same direction for each individual, instead highlighting regions where change took place within individuals regardless of direction. Exploring these individual patterns of change may help unpack the variable findings in the literature (Dichter et al., 2015; Fonseka et al., 2018).

Our study had several limitations. First, the approach we utilized only considered the relative contribution of the included sources of variance and therefore could not indicate if additional sources may have been missed. For simplicity, we examined only a limited number of categorical variables, but future research could include additional (continuous) factors (e.g., age; Geerligs et al., 2015). Similarly, it would be of interest to examine variability in dynamic functional connectivity as well. Second, our study would have benefitted from longer fMRI recordings. While previous studies indicated that the amount of data we had provided acceptable reliability for whole-brain FC estimates (Laumann et al., 2015; Gordon et al., 2017), several factors may influence such reliability (e.g., scanner, voxels size; Gratton et al., 2020), and we were not able to test this in our data directly. In addition, recent studies suggest even larger amounts of data (e.g., up to 200 min per participant) are needed to achieve high reliability of individual connectivity estimates, especially for single connections and noncortical regions (Gratton et al., 2020). Relatedly, a detailed examination of the sources of variance in fMRI data and the impact of different preprocessing pipelines would be warranted. In addition, like most research in psychology, our sample and we as researchers came from “WEIRD” (Western, Educated, Industrialized, Rich and Democratic) populations that represent only ∼12% of humanity (Henrich et al., 2010), indicating the need to invest in research done with and by more diverse people. Lastly, the use of biological sex and how it was measured was suboptimal. Namely, sex is not binary and other relevant, interrelated factors, such as gender expression and gender identity, were not accounted for, which may have led to measurement errors (Lindqvist et al., 2021). However, given the indications of the importance of sex and gender in the context of antidepressant treatment, we decided to use the information available to us.

Here we show that common and individual-specific patterns of functional connectivity explain most of the variance in FC data, while more commonly reported group-specific patterns account for only a small amount. To our knowledge, no other studies have quantified the relative contributions of individual and group-level variation in neuroimaging data in the context of MD and antidepressant treatment. More research will be needed to determine how the interplay between individual and group-common features of individual brain networks can help with better identifying and treating MD.

## Synthesis

Reviewing Editor: Jonathan Lee, University of Birmingham Decisions are customarily a result of the Reviewing Editor and the peer reviewers coming together and discussing their recommendations until a consensus is reached. When revisions are invited, a fact-based synthesis statement explaining their decision and outlining what is needed to prepare a revision will be listed below. The following reviewer(s) agreed to reveal their identity: Brian Kraus, Joan Guàrdia-Olmos.

There remain a few outstanding queries, linked to the original reviews and the response/revisions provided. The following is the full review, to which a further response is requested.

I again thank the authors for responding to my previous reviewer comments and addressing some of my concerns. I especially appreciate them adding additional information about the methods of the study and performing the analysis I requested comparing each individual's similarity to themselves versus that of the most similar participant. However, unfortunately I must admit that in several other ways I found this revision to be disappointing. In response to several of my other comments, I feel that the authors chose to make minor cosmetic changes to the text rather than empirically address any of the concerns noted. Specifically, my previous reviewer comments about the authors not assessing the reliability of their own data and performing sensitivity analyses on their processing pipeline have not been adequately addressed in my opinion. I have noted these concerns below taking into account the authors' responses, as well as some other minor issues.

-I think the biggest issue I still have with this revision is that many of its assumptions (e.g., the reported power analysis) and conclusions are based on the notion that the data here have similar reliability to past reports (e.g., Gordon et al., 2017; Laumann et al., 2015). But, this same level of reliability has not actually been demonstrated in the reported results here. In their reviewer responses, the authors state that because of the lack of data available (~90 minutes per subject), a split-half reliability analysis is impossible to perform here. However, this has been empirically demonstrated not to be the case. As is reported in Gordon et al. (2017) and Laumann et al. (2015), the reliability of functional connectivity estimates starts to plateau around 40 minutes. This means that for the purposes of a split-half reliability analysis, having 40 minutes of data in each split-half is very similar to having up to hundreds of minutes of data in each split-half. Furthermore, reliability curves look very similar with substantially less data in each split-half, even when the data is sequentially sampled and not randomly sampled from a larger quantity of data (see MSC09 in Figure 2A of Kraus et al., 2021). Due to this substantial evidence that this analysis is feasible, and the centrality of the reliability of the data to the authors' methods and conclusions, I do not think it is unreasonable for the authors to report their own split-half reliability analysis on the data in their study.

-A related issue to the reliability analysis mentioned above is my comment on the original paper about the motion correction strategy used here. While I appreciate the authors' revision clarifying their methods overall and their discussion of how their motion correction strategy is similar to censoring, the authors' rationale for interpolating censored frames appears to be that they were considering reporting dynamic connectivity analyses that require the full timeseries. Additionally, they state that the best practices for motion correction are not settled, which I wholeheartedly agree with. However, one of the best practices for evaluating the robustness of a finding in neuroimaging is to see if it is robust to different processing pipelines (Poldrack et al., 2017). Therefore, I think it is fairly reasonable for the authors to attempt to replicate their analyses without interpolating data frames, as the static connectivity analyses they report in this paper do not require uncensored timeseries. Alternatively, as I proposed in my previous comment, I think it is also adequate to just use the number of censored frames as a covariate in subsequent statistical analyses to reduce the analytic burden on the authors.

-I apologize that in my previous comments I did not realize that the "shuffle.m" function is native to MATLAB. However, the "fdr.m" function called upon by the authors does not appear to be a native MATLAB function, and thus I do not believe the authors' code will run without including it.

-This is a minor point I noticed upon reading the updated methods section, but the authors appear to run their task models on the surface after the data were smoothed. This is a slight deviation from Gratton et al. (2018), where the task modeling was done in the volume before smoothing. I doubt this will significantly impact the results as removing task activations has only a modest effect on connectivity estimates, but this difference in processing from Gratton et al. (2018) may be something the authors want to note.

References:

Gordon, E. M., Laumann, T. O., Gilmore, A. W., Newbold, D. J., Greene, D. J., Berg, J. J., Ortega, M., Hoyt-Drazen, C., Gratton, C., Sun, H., Jacqueline, H. M., Coalson, R. S., Nguyen, A. L., McDermott, K. B., Shimony, J. S., Snyder, A. Z., Schlaggar, B. L., Petersen, S. E., &Dosenbach, N. U. (2017). Precision functional mapping of individual human brains. Neuron, 95(4), 791-807.

Gratton, C., Laumann, T. O., Nielsen, A. N., Greene, D. J., Gordon, E. M., Gilmore, A. W., Nelson, S. M., Coalson, R. S., Snyder, A. Z., &Schlaggar, B. L. (2018). Functional brain networks are dominated by stable group and individual factors, not cognitive or daily variation. Neuron, 98(2), 439-452.

Kraus, B. T., Perez, D., Ladwig, Z., Seitzman, B. A., Dworetsky, A., Petersen, S. E., &Gratton, C. (2021). Network variants are similar between task and rest states. NeuroImage, 229, 117743.

Laumann, T. O., Gordon, E. M., Adeyemo, B., Snyder, A. Z., Joo, S. J., Chen, M.-Y., Gilmore, A. W., McDermott, K. B., Nelson, S. M., &Dosenbach, N. U. (2015). Functional system and areal organization of a highly sampled individual human brain. Neuron, 87(3), 657-670.

Poldrack, R. A., Baker, C. I., Durnez, J., Gorgolewski, K. J., Matthews, P. M., Munafò, M. R., Nichols, T. E., Poline, J.-B., Vul, E., &Yarkoni, T. (2017). Scanning the horizon: Towards transparent and reproducible neuroimaging research. Nature Reviews Neuroscience, 18(2), 115-126.
